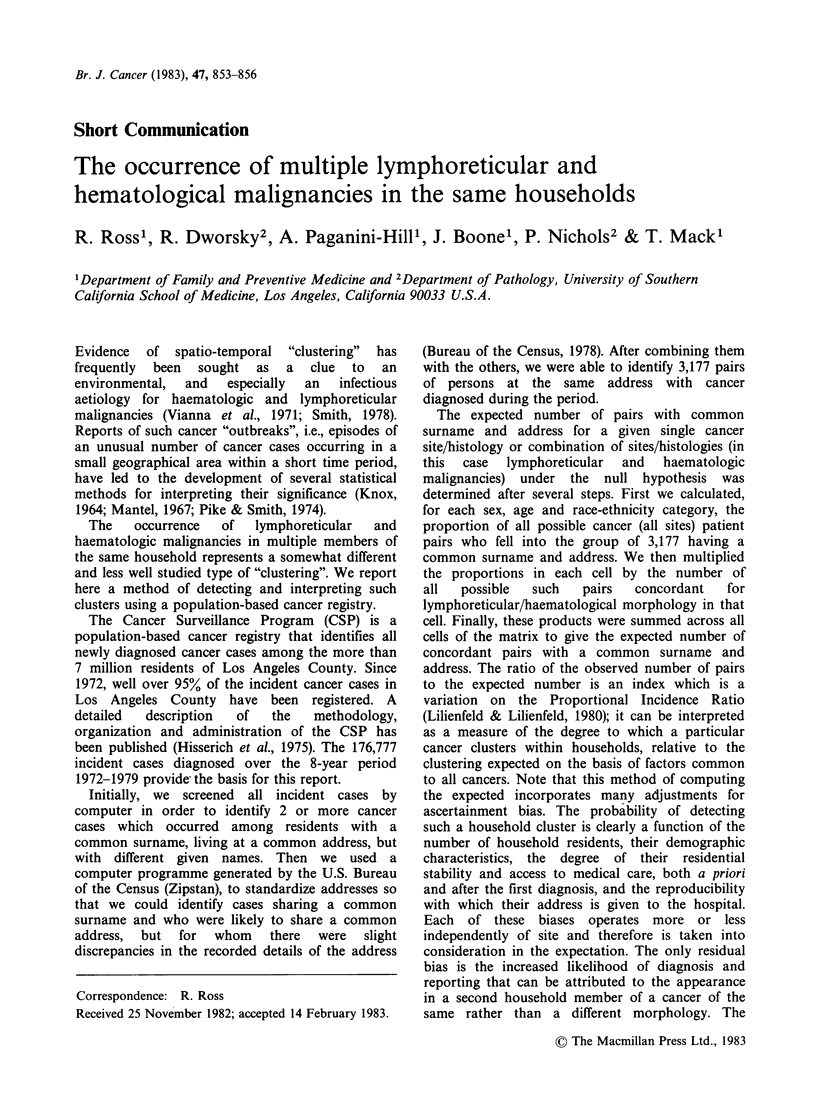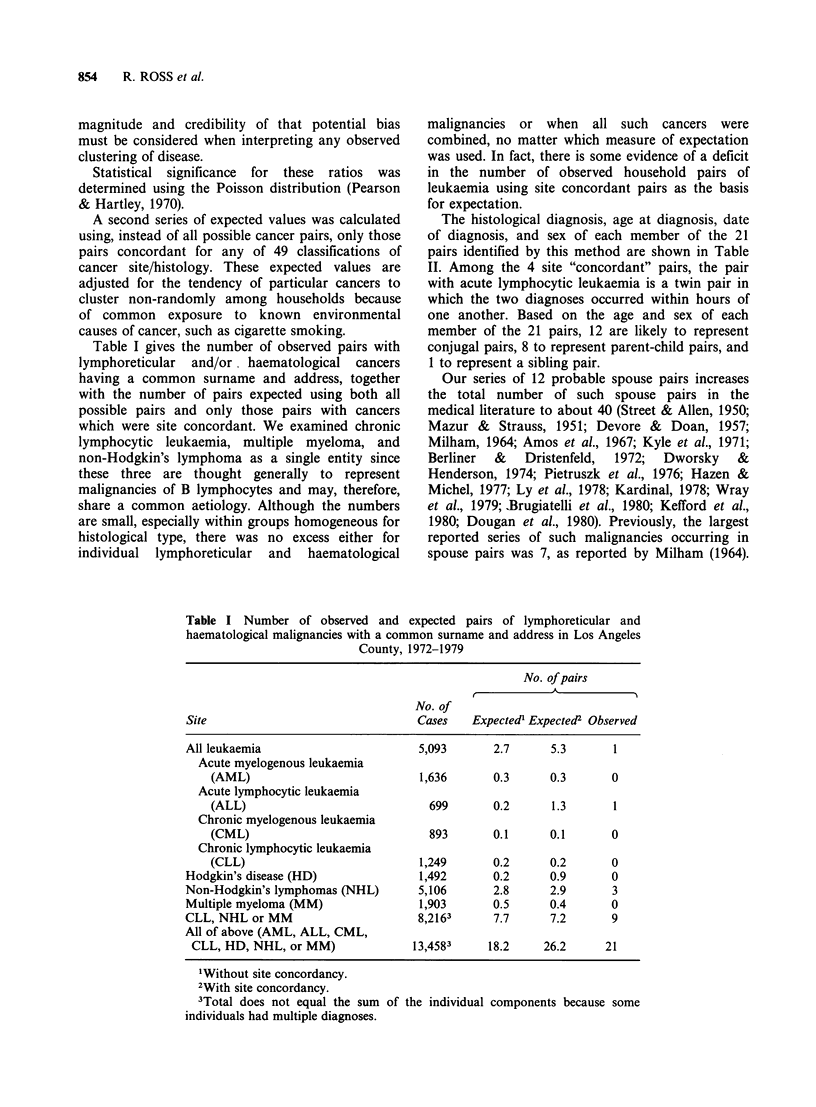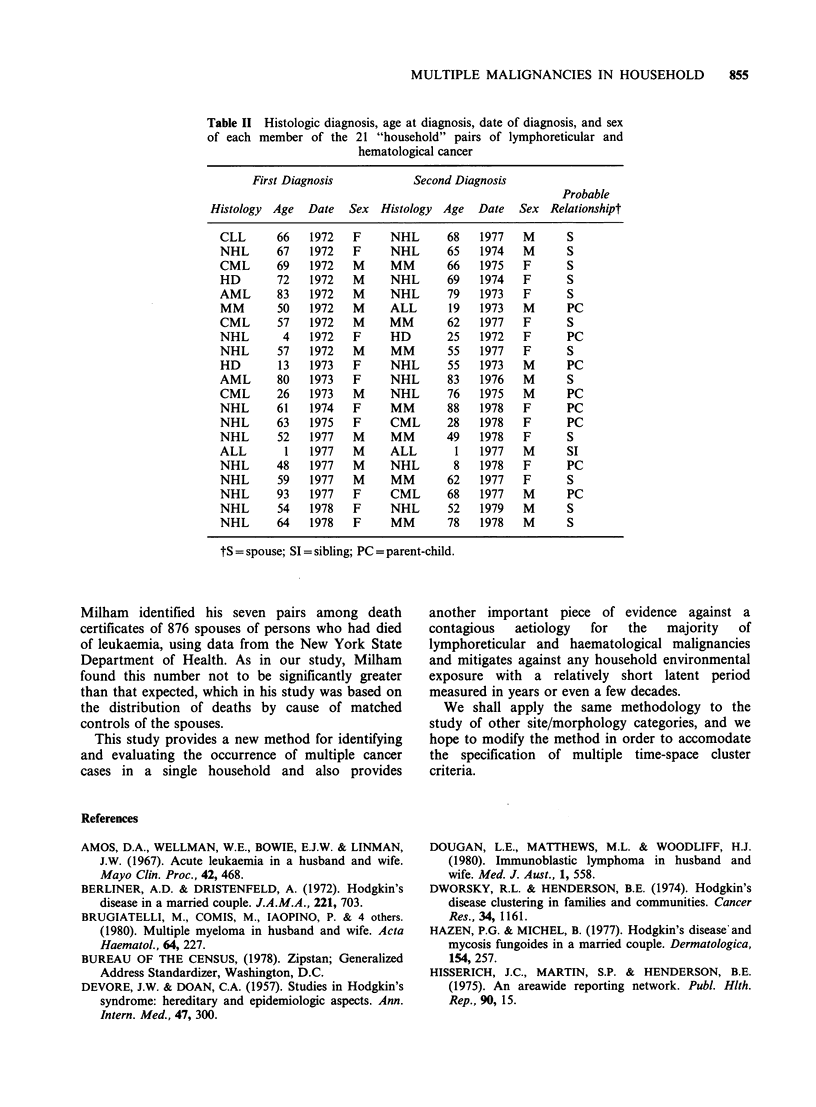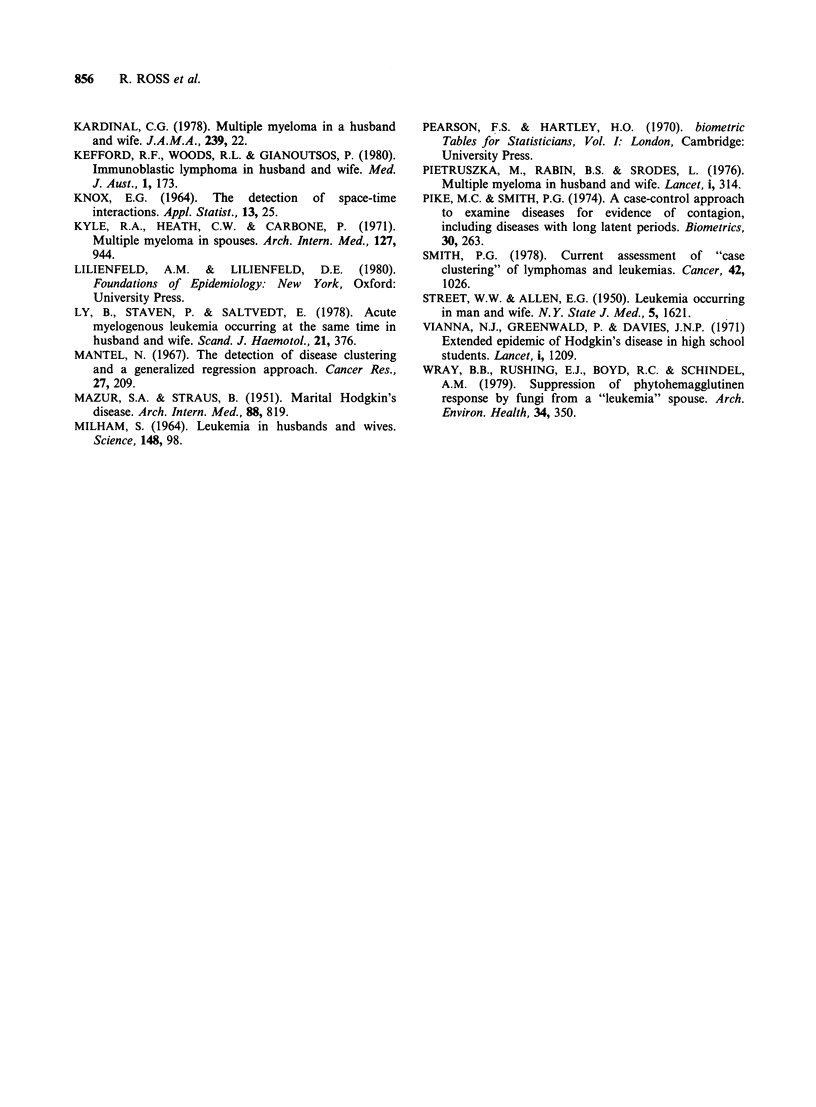# The occurrence of multiple lymphoreticular and hematological malignancies in the same households.

**DOI:** 10.1038/bjc.1983.141

**Published:** 1983-06

**Authors:** R. Ross, R. Dworsky, A. Paganini-Hill, J. Boone, P. Nichols, T. Mack


Br. J. Cancer (1983), 47, 853–856

### Short Communication

### The occurrence of multiple lymphoreticular and hematological malignancies in the same households

R. Ross[1], R. Dworsky[2], A. Paganini-Hill[1], J. Boone[1], P. Nichols[2] & T. Mack[1]

[1]*Department of Family and Preventive Medicine and* [2]*Department of Pathology, University of Southern California School of Medicine, Los Angeles, California 90033 U.S.A.*

Evidence of spatio-temporal "clustering" has frequently been sought as a clue to an environmental, and especially an infectious aetiology for haematologic and lymphoreticular malignancies (Vianna *et al.*, 1971; Smith, 1978). Reports of such cancer "outbreaks", i.e., episodes of an unusual number of cancer cases occurring in a small geographical area within a short time period, have led to the development of several statistical methods for interpreting their significance (Knox, 1964; Mantel, 1967; Pike & Smith, 1974).

The occurrence of lymphoreticular and haematologic malignancies in multiple members of the same household represents a somewhat different and less well studied type of "clustering". We report here a method of detecting and interpreting such clusters using a population-based cancer registry.

The Cancer Surveillance Program (CSP) is a population-based cancer registry that identifies all newly diagnosed cancer cases among the more than 7 million residents of Los Angeles County. Since 1972, well over 95% of the incident cancer cases in Los Angeles County have been registered. A detailed description of the methodology, organization and administration of the CSP has been published (Hisserich *et al.*, 1975). The 176,777 incident cases diagnosed over the 8-year period 1972–1979 provide the basis for this report.

Initially, we screened all incident cases by computer in order to identify 2 or more cancer cases which occurred among residents with a common surname, living at a common address, but with different given names. Then we used a computer programme generated by the U.S. Bureau of the Census (Zipstan), to standardize addresses so that we could identify cases sharing a common surname and who were likely to share a common address, but for whom there were slight discrepancies in the recorded details of the address (Bureau of the Census, 1978). After combining them with the others, we were able to identify 3,177 pairs of persons at the same address with cancer diagnosed during the period.

The expected number of pairs with common surname and address for a given single cancer site/histology or combination of sites/histologies (in this case lymphoreticular and haematologic malignancies) under the null hypothesis was determined after several steps. First we calculated, for each sex, age and race-ethnicity category, the proportion of all possible cancer (all sites) patient pairs who fell into the group of 3,177 having a common surname and address. We then multiplied the proportions in each cell by the number of all possible such pairs concordant for lymphoreticular/haematological morphology in that cell. Finally, these products were summed across all cells of the matrix to give the expected number of concordant pairs with a common surname and address. The ratio of the observed number of pairs to the expected number is an index which is a variation on the Proportional Incidence Ratio (Lilienfeld & Lilienfeld, 1980); it can be interpreted as a measure of the degree to which a particular cancer clusters within households, relative to the clustering expected on the basis of factors common to all cancers. Note that this method of computing the expected incorporates many adjustments for ascertainment bias. The probability of detecting such a household cluster is clearly a function of the number of household residents, their demographic characteristics, the degree of their residential stability and access to medical care, both *a priori* and after the first diagnosis, and the reproducibility with which their address is given to the hospital. Each of these biases operates more or less independently of site and therefore is taken into consideration in the expectation. The only residual bias is the increased likelihood of diagnosis and reporting that can be attributed to the appearance in a second household member of a cancer of the same rather than a different morphology. The

Correspondence: R. Ross

Received 25 November 1982; accepted 14 February 1983.

© The Macmillan Press Ltd., 1983

magnitude and credibility of that potential bias must be considered when interpreting any observed clustering of disease.

Statistical significance for these ratios was determined using the Poisson distribution (Pearson & Hartley, 1970).

A second series of expected values was calculated using, instead of all possible cancer pairs, only those pairs concordant for any of 49 classifications of cancer site/histology. These expected values are adjusted for the tendency of particular cancers to cluster non-randomly among households because of common exposure to known environmental causes of cancer, such as cigarette smoking.

Table I gives the number of observed pairs with lymphoreticular and/or haematological cancers having a common surname and address, together with the number of pairs expected using both all possible pairs and only those pairs with cancers which were site concordant. We examined chronic lymphocytic leukaemia, multiple myeloma, and non-Hodgkin's lymphoma as a single entity since these three are thought generally to represent malignancies of B lymphocytes and may, therefore, share a common aetiology. Although the numbers are small, especially within groups homogeneous for histological type, there was no excess either for individual lymphoreticular and haematological malignancies or when all such cancers were combined, no matter which measure of expectation was used. In fact, there is some evidence of a deficit in the number of observed household pairs of leukaemia using site concordant pairs as the basis for expectation.

The histological diagnosis, age at diagnosis, date of diagnosis, and sex of each member of the 21 pairs identified by this method are shown in Table II. Among the 4 site "concordant" pairs, the pair with acute lymphocytic leukaemia is a twin pair in which the two diagnoses occurred within hours of one another. Based on the age and sex of each member of the 21 pairs, 12 are likely to represent conjugal pairs, 8 to represent parent-child pairs, and 1 to represent a sibling pair.

Our series of 12 probable spouse pairs increases the total number of such spouse pairs in the medical literature to about 40 (Street & Allen, 1950; Mazur & Strauss, 1951; Devore & Doan, 1957; Milham, 1964; Amos *et al.*, 1967; Kyle *et al.*, 1971; Berliner & Dristenfeld, 1972; Dworsky & Henderson, 1974; Pietruszk *et al.*, 1976; Hazen & Michel, 1977; Ly *et al.*, 1978; Kardinal, 1978; Wray *et al.*, 1979; Brugiatelli *et al.*, 1980; Kefford *et al.*, 1980; Dougan *et al.*, 1980). Previously, the largest reported series of such malignancies occurring in spouse pairs was 7, as reported by Milham (1964).

**Table I** Number of observed and expected pairs of lymphoreticular and haematological malignancies with a common surname and address in Los Angeles County, 1972–1979

| Site | No. of Cases | *No. of pairs* Expected[1] | Expected[2] | Observed |
|---|---|---|---|---|
| All leukaemia | 5,093 | 2.7 | 5.3 | 1 |
| Acute myelogenous leukaemia (AML) | 1,636 | 0.3 | 0.3 | 0 |
| Acute lymphocytic leukaemia (ALL) | 699 | 0.2 | 1.3 | 1 |
| Chronic myelogenous leukaemia (CML) | 893 | 0.1 | 0.1 | 0 |
| Chronic lymphocytic leukaemia (CLL) | 1,249 | 0.2 | 0.2 | 0 |
| Hodgkin's disease (HD) | 1,492 | 0.2 | 0.9 | 0 |
| Non-Hodgkin's lymphomas (NHL) | 5,106 | 2.8 | 2.9 | 3 |
| Multiple myeloma (MM) | 1,903 | 0.5 | 0.4 | 0 |
| CLL, NHL or MM | 8,216[3] | 7.7 | 7.2 | 9 |
| All of above (AML, ALL, CML, CLL, HD, NHL, or MM) | 13,458[3] | 18.2 | 26.2 | 21 |

[1]Without site concordancy.
[2]With site concordancy.
[3]Total does not equal the sum of the individual components because some individuals had multiple diagnoses.

**Table II** Histologic diagnosis, age at diagnosis, date of diagnosis, and sex of each member of the 21 "household" pairs of lymphoreticular and hematological cancer

| *First Diagnosis* | | | | *Second Diagnosis* | | | | |
|---|---|---|---|---|---|---|---|---|
| *Histology* | *Age* | *Date* | *Sex* | *Histology* | *Age* | *Date* | *Sex* | *Probable Relationship*† |
| CLL | 66 | 1972 | F | NHL | 68 | 1977 | M | S |
| NHL | 67 | 1972 | F | NHL | 65 | 1974 | M | S |
| CML | 69 | 1972 | M | MM | 66 | 1975 | F | S |
| HD | 72 | 1972 | M | NHL | 69 | 1974 | F | S |
| AML | 83 | 1972 | M | NHL | 79 | 1973 | F | S |
| MM | 50 | 1972 | M | ALL | 19 | 1973 | M | PC |
| CML | 57 | 1972 | M | MM | 62 | 1977 | F | S |
| NHL | 4 | 1972 | F | HD | 25 | 1972 | F | PC |
| NHL | 57 | 1972 | M | MM | 55 | 1977 | F | S |
| HD | 13 | 1973 | F | NHL | 55 | 1973 | M | PC |
| AML | 80 | 1973 | F | NHL | 83 | 1976 | M | S |
| CML | 26 | 1973 | M | NHL | 76 | 1975 | M | PC |
| NHL | 61 | 1974 | F | MM | 88 | 1978 | F | PC |
| NHL | 63 | 1975 | F | CML | 28 | 1978 | F | PC |
| NHL | 52 | 1977 | M | MM | 49 | 1978 | F | S |
| ALL | 1 | 1977 | M | ALL | 1 | 1977 | M | SI |
| NHL | 48 | 1977 | M | NHL | 8 | 1978 | F | PC |
| NHL | 59 | 1977 | M | MM | 62 | 1977 | F | S |
| NHL | 93 | 1977 | F | CML | 68 | 1977 | M | PC |
| NHL | 54 | 1978 | F | NHL | 52 | 1979 | M | S |
| NHL | 64 | 1978 | F | MM | 78 | 1978 | M | S |

†S = spouse; SI = sibling; PC = parent-child.

Milham identified his seven pairs among death certificates of 876 spouses of persons who had died of leukaemia, using data from the New York State Department of Health. As in our study, Milham found this number not to be significantly greater than that expected, which in his study was based on the distribution of deaths by cause of matched controls of the spouses.

This study provides a new method for identifying and evaluating the occurrence of multiple cancer cases in a single household and also provides another important piece of evidence against a contagious aetiology for the majority of lymphoreticular and haematological malignancies and mitigates against any household environmental exposure with a relatively short latent period measured in years or even a few decades.

We shall apply the same methodology to the study of other site/morphology categories, and we hope to modify the method in order to accomodate the specification of multiple time-space cluster criteria.

### References

AMOS, D.A., WELLMAN, W.E., BOWIE, E.J.W. & LINMAN, J.W. (1967). Acute leukaemia in a husband and wife. *Mayo Clin. Proc.*, **42**, 468.

BERLINER, A.D. & DRISTENFELD, A. (1972). Hodgkin's disease in a married couple. *J.A.M.A.*, **221**, 703.

BRUGIATELLI, M., COMIS, M., IAOPINO, P. & 4 others. (1980). Multiple myeloma in husband and wife. *Acta Haematol.*, **64**, 227.

BUREAU OF THE CENSUS, (1978). Zipstan; Generalized Address Standardizer, Washington, D.C.

DEVORE, J.W. & DOAN, C.A. (1957). Studies in Hodgkin's syndrome: hereditary and epidemiologic aspects. *Ann. Intern. Med.*, **47**, 300.

DOUGAN, L.E., MATTHEWS, M.L. & WOODLIFF, H.J. (1980). Immunoblastic lymphoma in husband and wife. *Med. J. Aust.*, **1**, 558.

DWORSKY, R.L. & HENDERSON, B.E. (1974). Hodgkin's disease clustering in families and communities. *Cancer Res.*, **34**, 1161.

HAZEN, P.G. & MICHEL, B. (1977). Hodgkin's disease and mycosis fungoides in a married couple. *Dermatologica*, **154**, 257.

HISSERICH, J.C., MARTIN, S.P. & HENDERSON, B.E. (1975). An areawide reporting network. *Publ. Hlth. Rep.*, **90**, 15.

KARDINAL, C.G. (1978). Multiple myeloma in a husband and wife. *J.A.M.A.*, **239**, 22.

KEFFORD, R.F., WOODS, R.L. & GIANOUTSOS, P. (1980). Immunoblastic lymphoma in husband and wife. *Med. J. Aust.*, **1**, 173.

KNOX, E.G. (1964). The detection of space-time interactions. *Appl. Statist.*, **13**, 25.

KYLE, R.A., HEATH, C.W. & CARBONE, P. (1971). Multiple myeloma in spouses. *Arch. Intern. Med.*, **127**, 944.

LILIENFELD, A.M. & LILIENFELD, D.E. (1980). *Foundations of Epidemiology: New York*, Oxford: University Press.

LY, B., STAVEN, P. & SALTVEDT, E. (1978). Acute myelogenous leukemia occurring at the same time in husband and wife. *Scand. J. Haemotol.*, **21**, 376.

MANTEL, N. (1967). The detection of disease clustering and a generalized regression approach. *Cancer Res.*, **27**, 209.

MAZUR, S.A. & STRAUS, B. (1951). Marital Hodgkin's disease. *Arch. Intern. Med.*, **88**, 819.

MILHAM, S. (1964). Leukemia in husbands and wives. *Science*, **148**, 98.

PEARSON, F.S. & HARTLEY, H.O. (1970). *biometric Tables for Statisticians, Vol. I: London*, Cambridge: University Press.

PIETRUSZKA, M., RABIN, B.S. & SRODES, L. (1976). Multiple myeloma in husband and wife. *Lancet*, **i**, 314.

PIKE, M.C. & SMITH, P.G. (1974). A case-control approach to examine diseases for evidence of contagion, including diseases with long latent periods. *Biometrics*, **30**, 263.

SMITH, P.G. (1978). Current assessment of "case clustering" of lymphomas and leukemias. *Cancer*, **42**, 1026.

STREET, W.W. & ALLEN, E.G. (1950). Leukemia occurring in man and wife. *N.Y. State J. Med.*, **5**, 1621.

VIANNA, N.J., GREENWALD, P. & DAVIES, J.N.P. (1971) Extended epidemic of Hodgkin's disease in high school students. *Lancet*, **i**, 1209.

WRAY, B.B., RUSHING, E.J., BOYD, R.C. & SCHINDEL, A.M. (1979). Suppression of phytohemagglutinen response by fungi from a "leukemia" spouse. *Arch. Environ. Health*, **34**, 350.